# Supplementary material for: Data- and knowledge-derived functional landscape of human solute carriers
Source: Mol Syst Biol. 2025 May 12;21(6):599–631. doi: 10.1038/s44320-025-00108-2 (PMC12130315; doi:10.1038/s44320-025-00108-2)
Supplement: Supplementary file 1 — Table EV1 [file 44320_2025_108_MOESM1_ESM.docx]

Goldmann et al. Data- and knowledge-derived functional landscape of human solute carriers (2025)

Table EV1. Description of data sources used in survey of SLC superfamily-wide disease associations.

| data source | description |
| --- | --- |
| Open Targets Genetics (<https://genetics.opentargets.org/>)  25 Nov 2021/ Version 6 | Human genetics and genomics data for systematic drug target identification and prioritization; Collects common genetic variants; Includes GWAS Catalog, UKBB, FinnGen. There is a collection of common genetic variants and a variant to gene score (v2g) describes how confident a variant can be linked to the gene function. The score is based on GWAS reported lead variants expanded to potentially causal variants. The genes are ranked based on all available functional data. The range of the score is between 0 and 1, higher scores supply more evidence for a functional association. V2g scores were retrieved for 86 SLC genes. A threshold was applied at 20 percent quantile (~0.11) of the overall distribution to reduce noise and filter for good evidence. There was no data for four SLCs on the X-chromosome available. |
| IEU OpenGWAS project (<https://gwas.mrcieu.ac.uk/>)  Version 5.9.0 | A database of 214,068,546,020 genetic associations from 42,410 GWAS summary datasets; Source for qtl, eqtl, pqtl, mqtl data sets. |
| Genebass (<https://genebass.org/>)  Version 0.7.8-alpha | Source for exome-based association statistics; UKBiobank is the primary source; 281k individuals; 3700 traits; Gene-based and single-variant testing, Aggregates SNV scores in a region. |
| ClinVar (<https://www.ncbi.nlm.nih.gov/clinvar/>)  Data freeze 01.02.2022 | Archive for human variations and phenotypes with supporting evidence. |
| Genome Aggregation Database (gnomAD) (<https://gnomad.broadinstitute.org>)  Version 2.1.1 | Collection of exome and genome sequencing data from different projects; Includes allele frequencies and other summary statistics. |
| LitVar (<https://www.ncbi.nlm.nih.gov/CBBresearch/Lu/Demo/LitVar/>)  Data freeze 01.02.2022 | Retrieval of variant relevant information from the biomedical literature. |
| UniProt (<https://www.uniprot.org/>) | Resource of protein sequence and functional annotation. |
| Orphanet (<https://www.orpha.net/>)  Version 5.52.0 11.04.2022 | Portal for rare diseases/human genes/genetic phenotypes. |
| Prioriy Index (<http://pi.well.ox.ac.uk:3010/>)  Data from [PMID: 31253980](https://pubmed.ncbi.nlm.nih.gov/31253980/) | Framework to prioritize potential targets; Integrating trait associated GWAS data with genomic features (nearby genes, eQTL, HiC); Disease ontologies and network connectivity; 15000 genes evaluated for 30 immune related traits (e.g., Asthma, Gout). The Supplementary material provides 23 SLC’s among the 150 most important genes for 30 immune related traits. |
| Ensembl (<http://www.ensembl.org>)  Release 106 | Ensembl is a genome browser for vertebrate genomes; Supports research in comparative genomics, evolution, sequence variation, transcriptional regulation, gene annotation, alignments, regulatory function prediction and disease data collection. |
