# Supplementary material for: Data- and knowledge-derived functional landscape of human solute carriers
Source: Mol Syst Biol. 2025 May 12;21(6):599–631. doi: 10.1038/s44320-025-00108-2 (PMC12130315; doi:10.1038/s44320-025-00108-2)
Supplement: Supplementary file 2 — Table EV2 [file 44320_2025_108_MOESM2_ESM.docx]

Goldmann et al. Data- and knowledge-derived functional landscape of human solute carriers (2025)

Table EV2. Resources integrated in the SLC data- and knowledgebase.

| resource /  reference | version / date | used for | context |
| --- | --- | --- | --- |
| ChEBI | 229 | unification of substrate terms; ontology relationship (generalized querying and analyses); compound properties | knowledgebase / dashboard |
| ClinVar | 2022-02-01 | variant-trait associations | knowledgebase |
| [(Digles *et al*, 2024)](https://sciwheel.com/work/citation?ids=16649254&pre=&suf=&sa=0&dbf=0) |  | SLC-specific tool compounds | dashboard |
| EFO |  | unification of disease terms | landscape |
| Ensembl |  | gene model annotations | knowledgebase |
| [(Ferrada & Superti‑Furga, 2022)](https://sciwheel.com/work/citation?ids=14056998&pre=&suf=&sa=0&dbf=0) |  | fold annotation and structural similarities | knowledgebase / dashboard |
| [(Frommelt *et al*, 2024)](https://sciwheel.com/work/citation?ids=16992541&pre=&suf=&sa=0&dbf=0) |  | analysis of the SLC interactome | dashboard |
| Genebass | 0.7.8-alpha | variant-trait associations | knowledgebase |
| gnomAD | 2.1.1 | variant-trait associations | knowledgebase |
| GO |  | localization annotation; unification of disease terms | knowledgebase |
| HGNC | 2024-01-17 | SLC definition; gene-level annotations; identifier matching | knowledgebase |
| Human Protein Atlas | 23.0 | tissue-level gene expression [(Uhlén *et al*, 2015)](https://sciwheel.com/work/citation?ids=67390&pre=&suf=&sa=0&dbf=0); subcellular localization [(Thul *et al*, 2017)](https://sciwheel.com/work/citation?ids=3601803&pre=&suf=&sa=0&dbf=0) | knowledgebase |
| IEU OpenGWAS project | 5.9.0 | variant-trait associations | knowledgebase |
| LitVar | 2022-02-01 | variant-trait associations | knowledgebase |
| Mondo | 2024-06-04 | unification of disease terms; ontology relationship (generalized querying and analyses) | knowledgebase / dashboard |
| NCBI | 2023-10-09 | functional summaries; functionally relevant links to literature (GeneRIFs) | knowledgebase |
| Open Targets | 6 | variant-trait associations | knowledgebase |
| Orphanet | 5.52.0 | variant-trait associations | knowledgebase |
| Prioriy Index  [(Fang *et al*, 2019)](https://sciwheel.com/work/citation?ids=7137760&pre=&suf=&sa=0&dbf=0) |  | variant-trait associations | knowledgebase |
| Protter |  | transmembrane topology visualizations | knowledgebase |
| UniProt | 2024_01 | protein-level annotations; localization annotation; variant-trait associations | knowledgebase |
| [(Wiedmer *et al*, 2024)](https://sciwheel.com/work/citation?ids=16992529&pre=&suf=&sa=0&dbf=0) |  | transcriptomics & metabolomics analyses of SLC overexpression cell lines | dashboard |
| [(Wolf *et al*, 2024)](https://sciwheel.com/work/citation?ids=17024398&pre=&suf=&sa=0&dbf=0) |  | genetic screens on SLC single and double knockout cell lines | dashboard |
| this study |  | manual curation of SLC substrates; SLC substrate classification; immunofluorescence images of SLC overexpression cell lines; subcellular localization annotation; SLC tree layout | knowledgebase / dashboard |
