# Supplementary material for: Data- and knowledge-derived functional landscape of human solute carriers
Source: Mol Syst Biol. 2025 May 12;21(6):599–631. doi: 10.1038/s44320-025-00108-2 (PMC12130315; doi:10.1038/s44320-025-00108-2)
Supplement: Supplementary file 11 — Expanded View Figures [file 44320_2025_108_MOESM11_ESM.pdf]

Expanded View Figures

Figure EV1. SLC expression and substrate annotation.

(**A**) Number of SLC genes expressed in relation to the total number of protein-coding genes expressed in 1206 human cell lines (data from (Uhlén et al, 2015)). The dotted line indicates the overall average of 256 expressed SLCs per cell line (56.2% of the 455 SLCs with expression data). The dashed line indicates the average of 2.2% of SLC genes among all expressed protein-coding genes in a cell line. Cell lines of selected origin are indicated by color, and the cell lines employed in the RESOLUTE consortium are labeled. (**B**) Number of SLC genes expressed in relation to the total number of protein-coding genes expressed in 50 human tissues (data from (Uhlén et al, 2015)). The dotted line indicates the overall average of 300 expressed SLCs per tissue (65.9% of the 455 SLCs with expression data). The dashed line indicates the average of 2.2% of SLC genes among all expressed protein-coding genes in a tissue. Tissue groups, as defined in the original data, are indicated by color and selected tissues deviating from the overall distribution are labeled. (**C**) Total number of SLC genes expressed in 36 tissue groups. The average is indicated by the dotted line. Classification of tissue specificity of gene expression is indicated by color (data from (Uhlén et al, 2015)). (**D**) Comparison of previous (Meixner et al, 2020) and updated SLC substrate classifications with regards to the terminology and membership of SLCs. The width of the connecting lines corresponds to the number of co-classified SLCs. (**E**) The proportion of SLC families for each substrate class, i.e., number of respective family members compared to the total number of SLCs in each substrate class. The total numbers are given in parentheses.

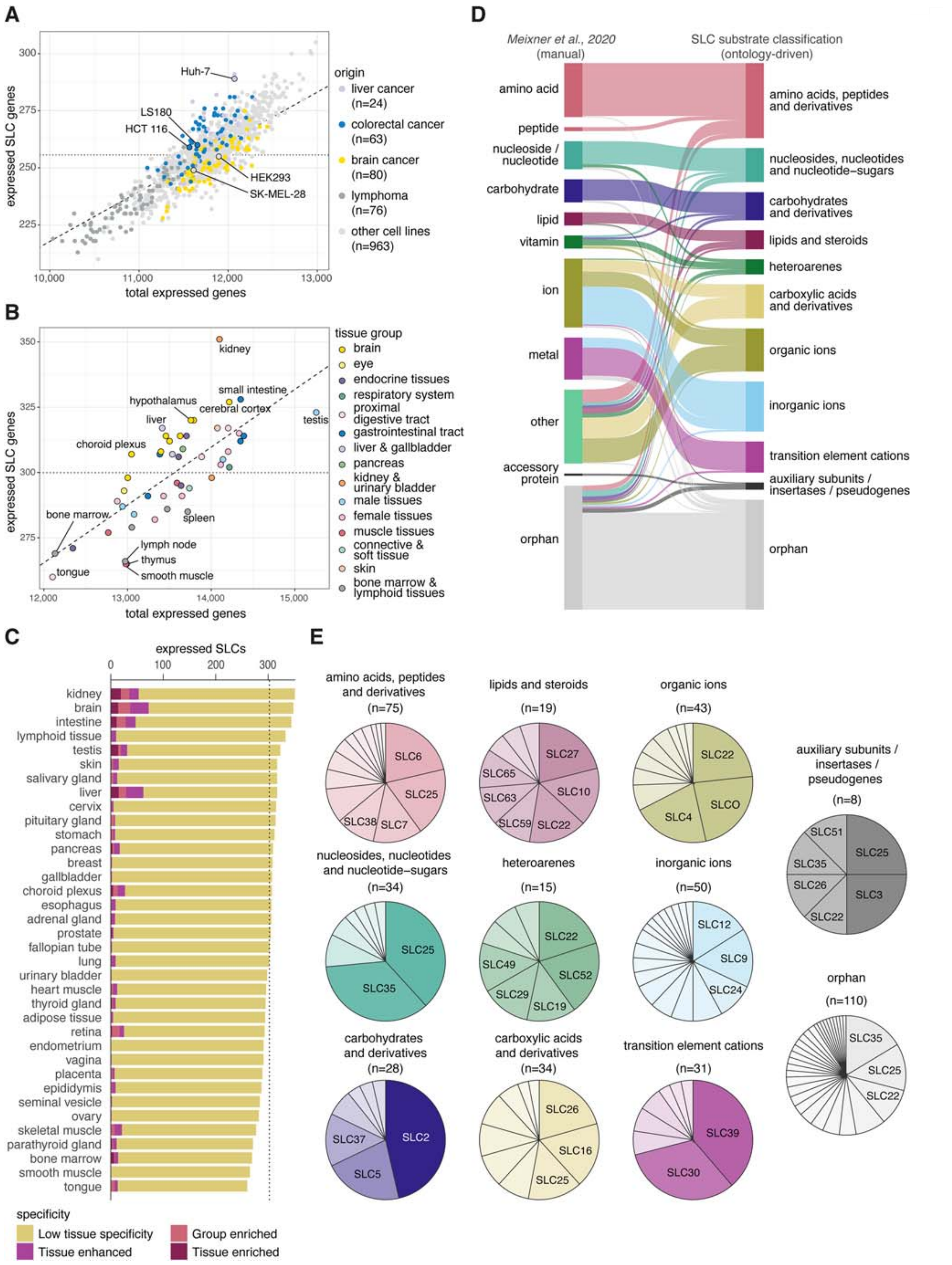
A
expressed SLC genes
total expressed genes
origin
liver cancer (n=24)
colorectal cancer (n=63)
brain cancer (n=80)
lymphoma (n=76)
other cell lines (n=963)
Huh-7
LS180
HCT 116
HEK293
SK-MEL-28
B
tissue group
brain
eye
endocrine tissues
respiratory system
proximal digestive tract
gastrointestinal tract
liver & gallbladder
pancreas
kidney & urinary bladder
male tissues
female tissues
muscle tissues
connective & soft tissue
skin
bone marrow & lymphoid tissues
kidney
small intestine
hypothalamus
cerebral cortex
testis
liver
choroid plexus
bone marrow
spleen
lymph node
thymus
tongue
smooth muscle
C
expressed SLCs
kidney
brain
intestine
lymphoid tissue
testis
skin
salivary gland
liver
cervix
pituitary gland
stomach
pancreas
breast
gallbladder
choroid plexus
esophagus
adrenal gland
prostate
fallopian tube
lung
urinary bladder
heart muscle
thyroid gland
adipose tissue
retina
endometrium
vagina
placenta
epididymis
seminal vesicle
ovary
skeletal muscle
parathyroid gland
bone marrow
smooth muscle
tongue
specificity
Low tissue specificity
Group enriched
Tissue enhanced
Tissue enriched
D
Meixner et al., 2020 (manual)
SLC substrate classification (ontology-driven)
amino acid
peptide
nucleoside / nucleotide
carbohydrate
lipid
vitamin
ion
metal
other
accessory protein
orphan
amino acids, peptides and derivatives
nucleosides, nucleotides and nucleotide–sugars
carbohydrates and derivatives
lipids and steroids
heteroarenes
carboxylic acids and derivatives
organic ions
inorganic ions
transition element cations
auxiliary subunits / insertases / pseudogenes
orphan
E
amino acids, peptides and derivatives (n=75)
lipids and steroids (n=19)
organic ions (n=43)
auxiliary subunits / insertases / pseudogenes (n=8)
nucleosides, nucleotides and nucleotide–sugars (n=34)
heteroarenes (n=15)
inorganic ions (n=50)
orphan (n=110)
carbohydrates and derivatives (n=28)
carboxylic acids and derivatives (n=34)
transition element cations (n=31)

© The Author(s)

A

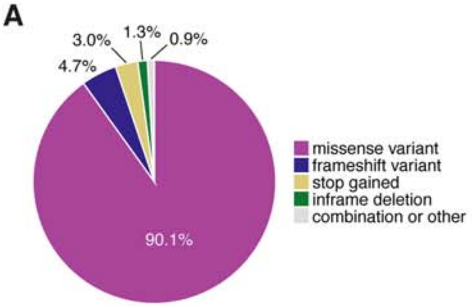

B

ontology of mapped trait	variant-level associations (pathogenic)	gene-level associations (pathogenic)	distinct gene-term associations (pathogenic)
MONDO	3,475 (3,244)	218 (216)	946 (780)
EFO	4,035 (0)	261 (0)	2,911 (0)
HP	303 (0)	5 (0)	245 (0)
GO	3 (0)	1 (0)	3 (0)
total	7,816 (3,244)	485 (216)	4,105 (780)

C

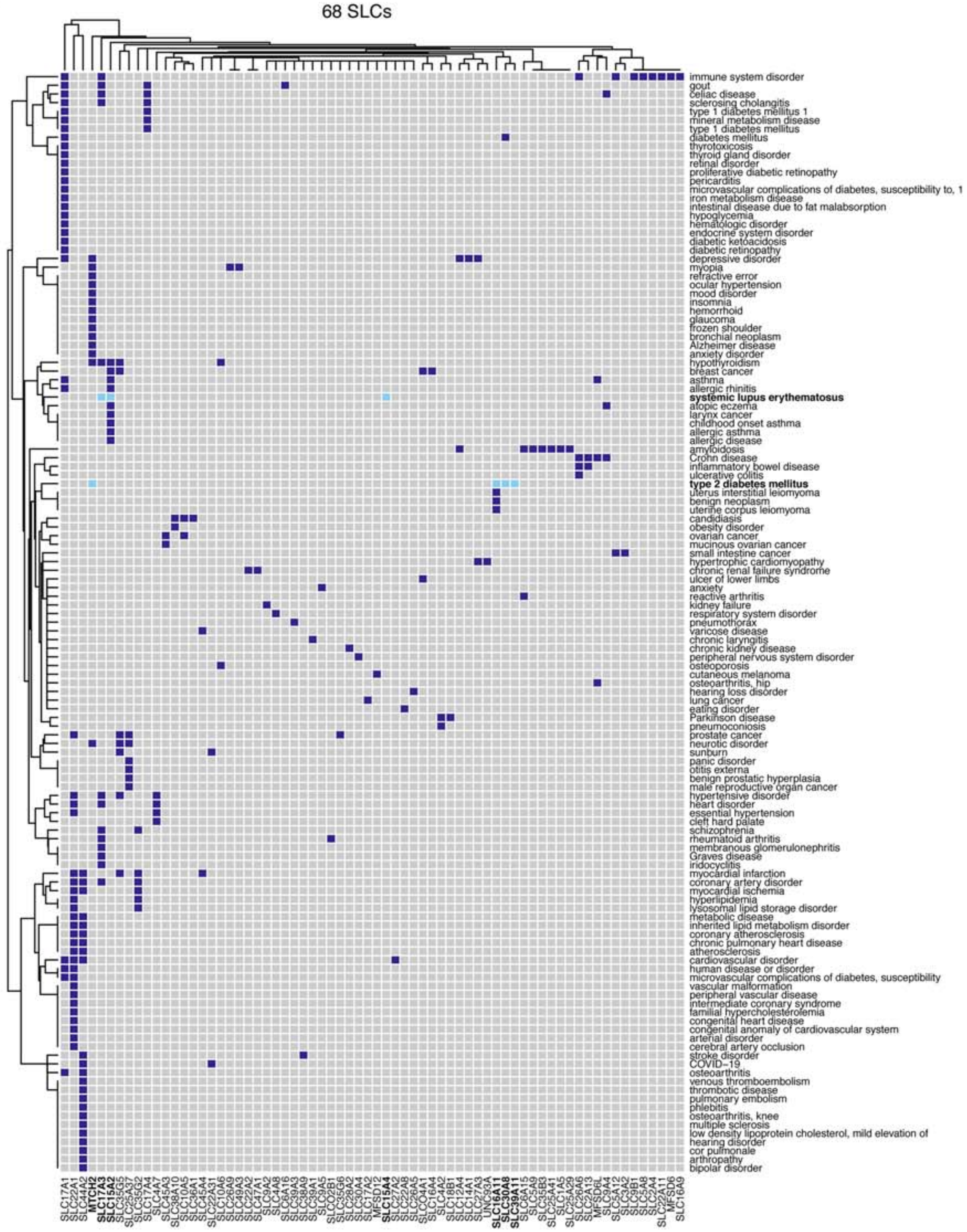

Figure EV2. Collection of SLC genetic variants and disease associations.

(**A**) Proportions of the main types of genetic alterations of variants with a protein-altering consequence on the canonical transcript of an SLC gene. (**B**) Overview of the number of variants with trait mapping to ontologies Mondo, EFO, HP and GO. Numbers in parentheses are pathogenic variants. (**C**) Heatmap of 68 SLCs without pathogenic associations in ClinVar and Orphanet (*x* axis) and novel 127 putative pathogenic terms (*y* axis). Examples discussed are marked in bold and light blue.

© The Author(s)

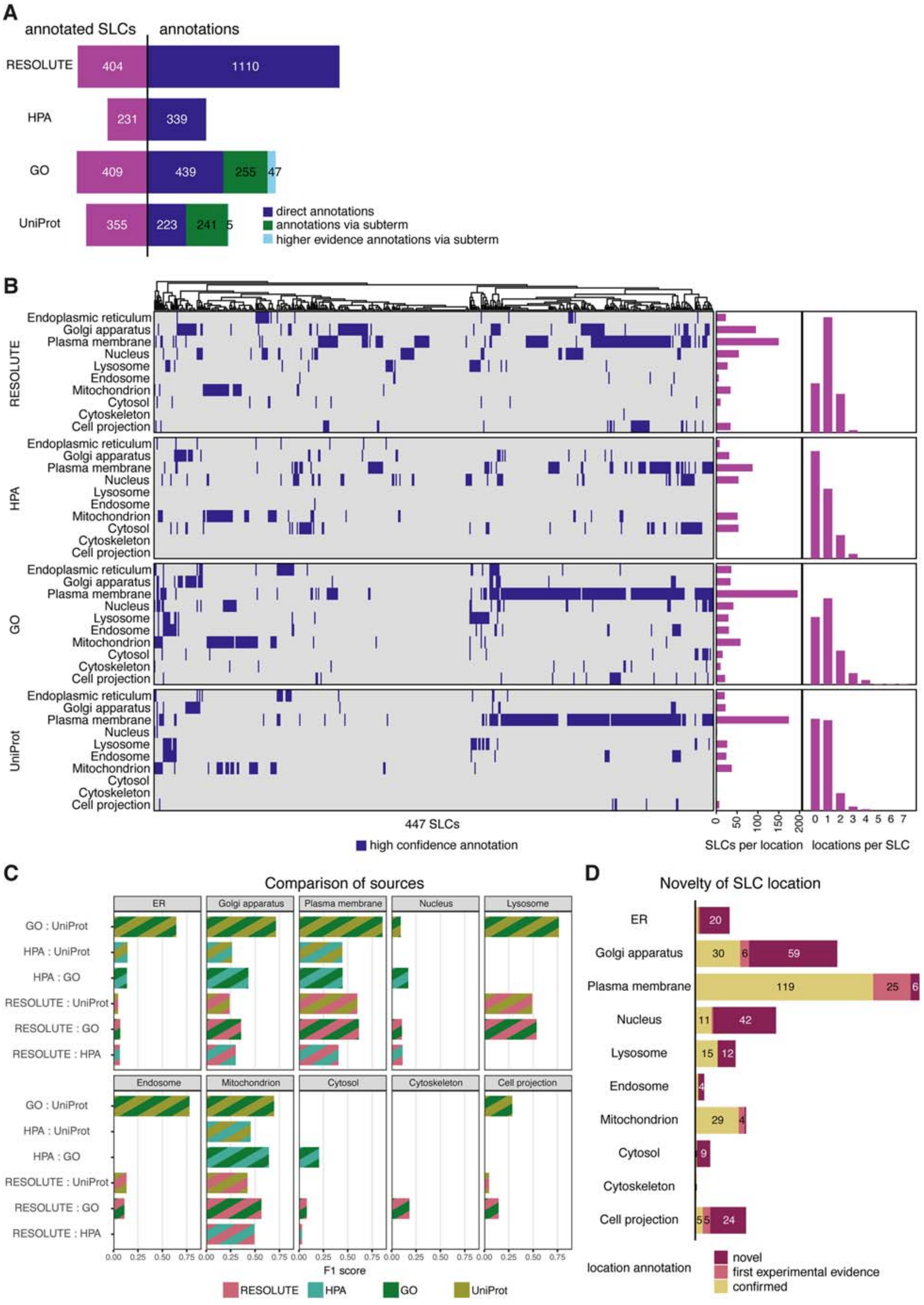
A
annotated SLCs
annotations
RESOLUTE
404
1110
HPA
231
339
GO
409
439
255
47
UniProt
355
223
241
5
direct annotations
annotations via subterm
higher evidence annotations via subterm
B
Endoplasmic reticulum
Golgi apparatus
Plasma membrane
Nucleus
Lysosome
Endosome
Mitochondrion
Cytosol
Cytoskeleton
Cell projection
RESOLUTE
HPA
GO
UniProt
447 SLCs
high confidence annotation
0 50 100 150 200
SLCs per location
0 1 2 3 4 5 6 7
locations per SLC
C
Comparison of sources
ER
Golgi apparatus
Plasma membrane
Nucleus
Lysosome
Endosome
Mitochondrion
Cytosol
Cytoskeleton
Cell projection
GO : UniProt
HPA : UniProt
HPA : GO
RESOLUTE : UniProt
RESOLUTE : GO
RESOLUTE : HPA
0.00 0.25 0.50 0.75
F1 score
RESOLUTE
HPA
GO
UniProt
D
Novelty of SLC location
ER
20
Golgi apparatus
30 6 59
Plasma membrane
119 25 6
Nucleus
11 42
Lysosome
15 12
Endosome
4
Mitochondrion
29 4
Cytosol
9
Cytoskeleton
Cell projection
5 5 24
location annotation
novel
first experimental evidence
confirmed

◀ **Figure EV3. Comparison of subcellular location annotations for the SLC superfamily.**

(**A**) Comparison of the number of location annotations by resource (as in Fig. 3C), highlighting the effect of the described ontology matching process (Methods). For GO and UniProt, a considerable number of annotations were only captured via subterms of the 10 selected locations. A smaller number of location annotations did match a selected location term but ended up with an increased evidence level via an additionally mapped subterm of the respective location. (**B**) Overview and comparison of all high-confidence location annotations across RESOLUTE, HPA, GO and UniProt. The histograms show the prevalence of annotations for each of the selected 10 locations per data source, as well as the distribution of the number of location annotations per SLC for each data source. (**C**) Scoring of the agreement in annotations per subcellular location, for each pair of annotation sources. The F1-score combines precision and recall between the two annotation sources, with a higher score corresponding to higher consistency. (**D**) Novelty of high-confidence experimental localization annotations by the RESOLUTE data set, for each of the 10 selected subcellular locations.

 © The Author(s)

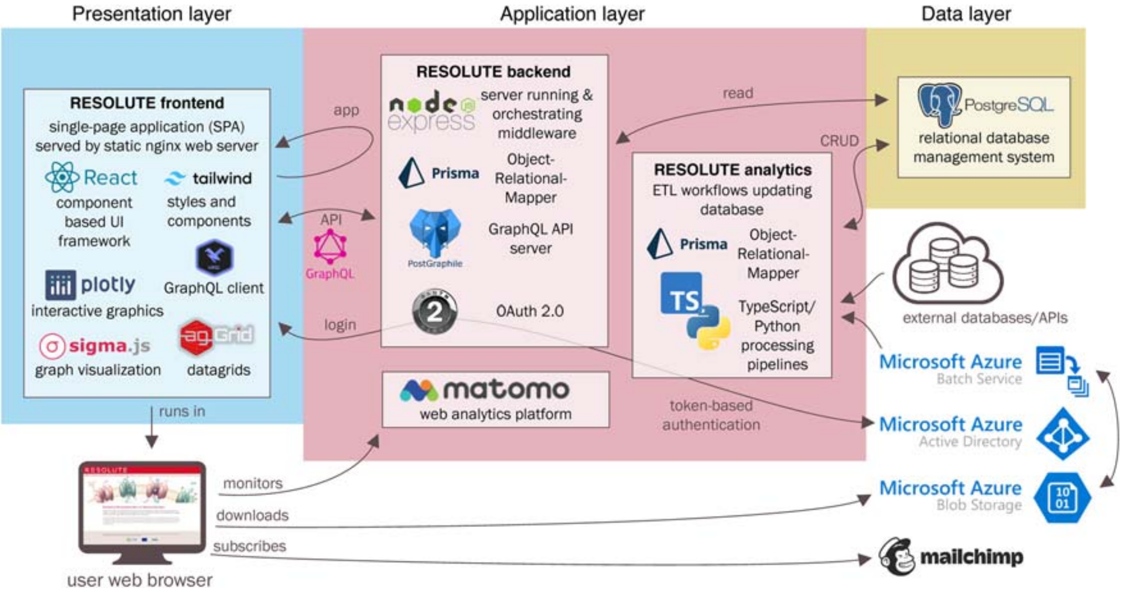

Figure EV4. The RESOLUTE web portal software architecture.

The web portal features a relational database handling the data layer, a backend and processing workflows handling the application layer, and a frontend in the form of a 'single-page' web application handling the presentation layer. Please refer to the Methods section for more details. UI user interface, API application programming interface, CRUD create, read, update, delete.

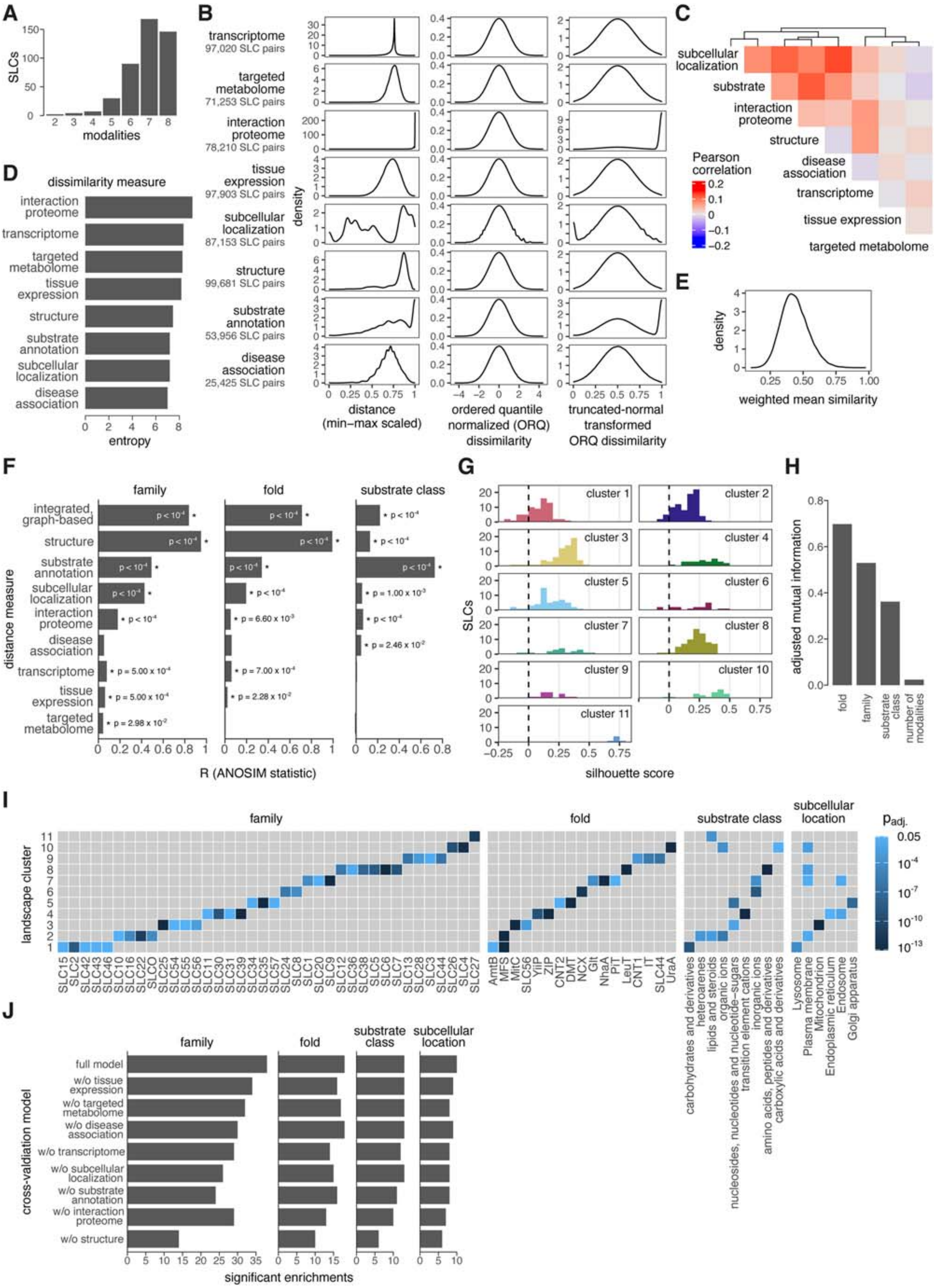
A
SLCs
modalities
B
transcriptome
97,020 SLC pairs
targeted metabolome
71,253 SLC pairs
interaction proteome
78,210 SLC pairs
tissue expression
97,903 SLC pairs
subcellular localization
87,153 SLC pairs
structure
99,681 SLC pairs
substrate annotation
53,956 SLC pairs
disease association
25,425 SLC pairs
density
distance (min–max scaled)
ordered quantile normalized (ORQ) dissimilarity
truncated-normal transformed ORQ dissimilarity
C
Pearson correlation
D
dissimilarity measure
entropy
E
weighted mean similarity
F
family
fold
substrate class
integrated, graph-based
distance measure
R (ANOSIM statistic)
G
SLCs
silhouette score
H
adjusted mutual information
number of modalities
I
landscape cluster
subcellular location
p adj.
carbohydrates and derivatives
heteroarenes
lipids and steroids
organic ions
nucleosides, nucleotides and nucleotide–sugars
transition element cations
inorganic ions
amino acids, peptides and derivatives
carboxylic acids and derivatives
Lysosome
Plasma membrane
Mitochondrion
Endoplasmic reticulum
Endosome
Golgi apparatus
J
full model
w/o tissue expression
w/o targeted metabolome
w/o disease association
w/o transcriptome
w/o subcellular localization
w/o substrate annotation
w/o interaction proteome
w/o structure
cross-validation model
significant enrichments

Figure EV5. Data sets used in constructing the SLC landscape and analysis of its clustering.

(**A**) Distribution of the number of modalities (data sets) available per SLC. (**B**) Distribution of SLC–SLC pair distances per modality. The original distributions of modality-specific distances are shown in the left column, scaled to the interval of [0,1] for visualization purposes. The central column shows the same distributions after transformation to a standard normal distribution using ordered quantile normalization. For distances with an upper or lower limit, values at these limits were excluded from this transformation. The right column shows the normalized distributions transformed to a truncated normal distribution, with the previously excluded values reintroduced at the corresponding boundary, resulting in dissimilarities comparable across modalities. (**C**) Correlations between the eight dissimilarities of all possible SLC–SLC pairs ($n = 99{,}681$), using Pearson's method on pairwise-complete observations. (**D**) Information content estimation for each of the eight dissimilarities, computed by the alpha entropy for continuous values. (**E**) Distribution of the overall SLC–SLC pair similarities, which resulted from subtracting the weighted average of up to eight dissimilarities for each pair from 1. (**F**) ANOSIM analysis for coherence of different distances (original distances and integrated landscape distance) with discrete SLC properties (family, fold, substrate class). Significant coherences ($P < 0.05$) are marked with an asterisk and the corresponding P value from a permutation-based test. Due to the test involving 10,000 iterations, P values below 10^{-4} cannot be accurately determined. (**G**) Distribution of silhouette scores of the members of the 11 different clusters derived from the SLC landscape. (**H**) Mutual information analysis of landscape clusters with discrete SLC properties. Fold, family, and substrate class all share considerable mutual information with the 11 clusters of the SLC landscape. The number of modalities does not correspond to cluster membership. (**I**) Results of the pairwise enrichment analysis of each SLC property level in each cluster, using Fisher's exact test. Only SLC property levels with significant enrichments are shown, colored by their multiple-testing corrected P values. (**J**) Summary of the enrichment analysis in panel I ('full model'), and additionally computed for SLC landscape clusterings generated by a leave-one-out cross-validation analysis. Models are ordered by the total number of enrichments per round of cross-validation. The full model not only shows the highest number of enrichments overall, but also in each SLC property analyzed.

© The Author(s)
